# Microbial contribution to spoilage of African breadfruit (*Artocarpus communis*, Forst) during storage

**DOI:** 10.1002/fsn3.28

**Published:** 2013-04-02

**Authors:** Olusegun B. Ajayi, Tinuola T. Adebolu

**Affiliations:** ^1^ Department of Botany and Microbiology University of Ibadan Ibadan Nigeria; ^2^ Department of Microbiology Federal University of Technology Akure Nigeria

**Keywords:** African breadfruit, different temperatures, morphological changes, spoilage, storage conditions

## Abstract

The contributions of microorganisms in the deterioration of African breadfruit during storage were investigated in this study. Matured fruits of the seedless variety of the African breadfruit (*Artocarpus communis*, Forst) were stored under different temperature conditions and morphological changes observed at 24‐h intervals for 120 h. Spoilage of breadfruit was observed after 72 h with microbial growth. Although all the fruits in the different media deteriorated by the 72nd hour (this was revealed in morphology and confirmed by the proximate analysis which showed an increase in %crude protein in all the stored fruits), microbial growth was observed only in those fruits stored at room temperature and in water, and there was no significant microbial growth in fruits stored in refrigerator, freezer, and vinegar. A higher rate of deterioration (i.e., higher %crude protein) was observed in morphology of fruits which had microbial growth during storage (i.e., those stored in the room, under water, and refrigerator) than in those stored fruits with no significant microbial growth. The difference between the %crude protein in fruits where there is microbial growth and that of the fruits where there is no microbial growth (i.e., freezer and vinegar) proved to be significant (*P *≤* *0.05). The study thus reveals that microorganisms play a substantial role in the spoilage of African breadfruit. A strain of the *Aspergillus* sp., two strains of the *Penicillium* sp., and a strain of the *Molinia* sp. were isolated as fungal spoilage organisms. *Bacillus* sp. and *Pseudomonas* sp. strains were isolated as bacteria spoilage organisms.

## Introduction

### Breadfruit

Breadfruit is an important energy food containing starch and sugars, especially common in the Pacifics (Secretariat of the Pacific Community [SPC] 2006). The levels of starch and sugars vary according to the stage of ripeness at which the fruit is eaten (SPC 2006). The Pacific varieties of breadfruit are reputed to be rich in vitamin C, thiamin, and the precursor of vitamin A – carotenoids (a fair source of vitamin B and niacin); the amount of these varies with ripeness. It is also rich in fiber and the mineral calcium (Ragone et al. 1996). It contains 5.3 g/100 g of protein (SPC 2006).

### African breadfruit varieties

The African breadfruit is of two varieties – the seeded (*Artocarpus altilis*) and the seedless (*Artocarpus communis*). These varieties of the African breadfruit contain on average a higher amount of dietary fiber and protein – 36 g/100 g and 6.1 g/100 g, respectively – than that of the Pacific (Ejidike and Ajileye 2007). Moreover, having compared the nutrient value of the African breadfruit with that of existing carbohydrates sources, such as yam, potato, and cassava, several scholars have concluded that African breadfruit (*A. communis*) have the nutrient potential to replace several of these carbohydrate sources in food consumed on the continent, especially flour‐based foods (Ravindram and Sivalcanessan 1995; Steve et al. 1995; Omobuwajo 2000; Ragone et al. 2006; Ugwu and Oranye 2006; Olaoye and Onilude 2008).

Olaoye et al. (2007) found that one quality of the African breadfruit that makes it a possible candidate for composite flour for bread making is its dough quality. The African breadfruit has thus been made into flour and evaluated in bakery products, including bread production from wheat–breadfruit composite flour (Olatunji and Akinrele 1978; Graham and DeBravo 1981; Olaoye and Onilude 2008). In another study, Olaoye et al. 2007 published some findings on the use of wheat–breadfruit composite flour in biscuit making. According to these authors, good‐quality and acceptable baked products could be derived from composite flours with up to certain levels of breadfruit flour substitution in wheat flour. Esuoso and Bamiro (1995) carried out some research findings on the use of composite flours of breadfruit–wheat in the production of baked products.

### The problem

The seedless variety of the African breadfruit is known to have problem of storage due to high respiration rate observed in the fruit during storage (Thompson et al. 1974; Wootton and Tumali 1984; Salveit 1996; Omobuwajo 2000). This problem of storage has been attributed to account for the loss through wastage of a great proportion of the breadfruit produced in West Africa especially in the western part of Nigeria due to poor postharvest handling (Pantastico 1975; Esuoso and Bamiro 1995; Suleiman 2002). Steve et al. (1995) reported that between 60% and 80% of breadfruit produced in the southwestern Nigeria is wasted as a result of deterioration and lack of use. Worell and Carrington (1997) reported that the respiration rate of stored breadfruit ranges between 94–564 mg CO_2_/kg per hour and 362–597 mg CO_2_/kg per hour. This was the reason for rapid deterioration of the fruits immediately after harvest, as concluded by Worell and Carrington (1997).

However, Omobuwajo and Wilcox (1989) reported that microorganisms which are associated with the spoilage of *A. communis* when on the field during planting include *Aspergillus* sp., *Rhizopus* sp., *Staphylococcus* sp., and *Mucor* sp. We have investigated in this study the spoilage microorganisms involved during storage of the African breadfruit and their contributions to spoilage.

## Materials and Methods

### Preparation of culture media

The media used for the isolation of the microorganism were Sabouraud's dextrose agar and nutrient agar. These agars were prepared according to manufacturer's instructions (BDH). The media were sterilized at a temperature of 121°C for 15 min in an autoclave.

### Preparation of the breadfruit samples

Twenty‐five healthy fruits were harvested at the matured, but not fully riped, stage from a breadfruit tree from a location in Akure, Ondo State, Nigeria. These fruits were washed in chlorinated water, rinsed with sterile water, and blotted dry with clean towel. Five of the fruits were cut, dried, and subjected to proximate and mineral content analyses, which included ash content, carbohydrate, crude fiber, crude fat, crude protein, and moisture compositions. This was to ascertain their proximate and mineral content from the field. Ten of the remaining fruits were cut into halves to observe the morphological changes and microbial growth in the pulp of the breadfruit as time progresses in the course of the study, whereas the remaining 10 fruits were left whole to observe the changes that occur on the bark of the fruit.

### Growth of microbial spoilage organisms

The fruits that were not dried in the oven were then divided into five groups of four fruits each. Each group comprised two whole fruits and four halves. The five groups were then kept under different storage conditions for 120 h as follows:

The first group was placed on sterile trays laid with aluminum foil and stored in a temperature‐controlled room at 28 ± 2°C. The second group was stored in water in a big water bath with temperature regulated and maintained at 20 ± 2°C. The third and fourth groups were kept in a refrigerator and freezer at temperatures of 10 ± 2°C and −6 ± 2°C. The last group was kept in a big jar of vinegar (10% acetic acid). After 120 h, the microbial load was determined and the isolation of spoilage organism determined.

### Isolation of spoilage organisms

Samples of fruits stored under different conditions were taken and prepared for microbiological examination through the method of Fawole and Oso (1998) and Sanni et al. (2002a,b). The surface of the pulp and bark and core were scraped into a Petri dish and prepared for examination by 10^−5^ dilution. The 10^−5^ dilution was used as the inoculum and it was plated out using pour plate method. The plates were then incubated at 37°C for 24 h for bacteria and at room temperature for 72 h for fungi. The pure microbial isolates were streaked onto new culture plates and incubated for 48 h.

### Preliminary identification tests

Various tests were carried out to identify and characterize the microorganisms that proliferate on the fruits during storage, according to Fawole and Oso (1998) and Sanni et al. (2002b). These tests include gram staining, motility tests, nitrate reduction, oxidase tests for bacteria, and microscopic morphological observations for fungal isolates.

### Proximate and mineral content analyses

**Figure 1:**
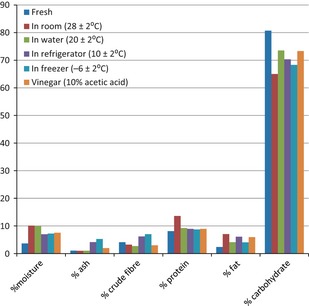
Proximate composition of breadfruit before and after storage for 120 h.

**Figure 2:**
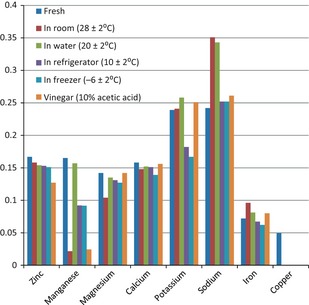
Mineral contents of breadfruit before and after storage for 120 h.

Standard proximate analyses methods were used according to Osundahunsi et al. (2003) and Ogunbanwo et al. (2008). Breadfruit flour was produced by drying using Gallenkamp OV‐160 laboratory oven drier and milling of the diced breadfruit using laboratory blender (Phillips). The moisture content was determined by oven‐drying weighed samples and calculations using the standard American Organisation for Analytical Chemistry (A.O.A.C.) methods (Figs. 1 and 2).

Other proximate analyses were carried out on the flour on dry matter basis according to conventional methods of A.O.A.C. as described by Ogunbanwo et al. (2008). Ash content was determined by dry ashing in a muffle furnace at 550°C, crude protein was determined using the micro‐Kjeldahl technique, crude fiber was determined as described by Kirk and Sawyer (1991), and crude fat was determined using a Soxhlet extractor (Tecato, Ikeja, Lagos, Nigeria) with petroleum ether. The %carbohydrate is calculated as the difference between the total mass of the breadfruit sample taken and the %protein, %fat and the %minerals analysed from the sample. All determinations were performed in triplicate.

Mineral content analysis such as iron, calcium, potassium, sodium, manganese, magnesium, and zinc was conducted on the ash solution of the breadfruit flour using atomic absorption spectrophotometer (Perkin Elmer 500 and Varian AA 475).

### Pathogenicity test

A test was conducted on fresh samples of fruits from the same breadfruit tree in the described location to confirm the pathogenicity of the organisms isolated from the stored fruits. This was performed by mixing a slightly turbid solution of the isolates in sterile water and inoculating the bark and pulps of the washed fresh fruits. The fruits were then placed under a laminar flow station at room temperature (i.e., 28 ± 2°C) for 120 h. At the expiration of the time, the inoculated fruits were checked for growth of the microorganisms.

### Statistical analysis

Data obtained were subjected to analysis of variance (ANOVA), the means being tested for significance at 5% level using Duncan's multiple range (DMR) test.

## Results and Discussion

A strain of the *Aspergillus* sp., two strains of the *Penicillium* sp., and a strain of the *Molinia* sp. were isolated as fungal spoilage organisms. *Bacillus* sp. and *Pseudomonas* sp. strains were isolated as bacteria spoilage organisms. These species of microorganisms were suspected after preliminary microscopy and biochemical tests were conducted (Table 1). However, molecular analyses of the strains to determine their genetic component and confirm their identity were not covered by the scope of this study.

**Table 1:** Preliminary identification tests for bacterial isolates from breadfruit (*Artocarpus communis*) stored for 120 h

Colony description	Shape	Gram staining	Motility	Spore	Oxidase	Oxygen tolerance	Nitrate	Lactose	Glucose	Maltose	Sucrose	Fructose	Mannitol	Arabinose	Raffinose	Temporary identity
Dirty brown and butyrous	Long rods	−	+	−	+	FA	+	−	−	−	−	−	−	−	−	*Pseudomonas* sp.
Creamy, spreading	Long rods	+	−	+	−	FA	+	−	A	A	A	−	A	AG	−	*Bacillus* sp.

A, ammonia produced; AG, ammonia with other gas (not confirmed) produced; FA, facultative aerobes.

Pathogenicity test revealed proliferation of the microorganisms inoculated on the fresh sample indicating their spoilage activities. The test thus proved positive.

In this study, it was observed that there was significant growth of microorganisms on the breadfruit stored at room temperature (28 ± 2°C) and in water at 20 ± 2°C, while no significant growth (*P *≤* *0.05) was observed for fruits stored in refrigerator, freezer at 10 ± 2°C and −6 ± 2°C, respectively; this was also true for vinegar (Table 2). It is interesting to note that morphological changes which indicate deterioration were observed in the fruits stored in the refrigerator, freezer, and vinegar (Table 3). This supports the established opinion that respiration causes spoilage in breadfruit. However, statistical analysis revealed significant difference between the deterioration observed in cases where microbial growth exists and where there was no growth. This is confirmed in the highest increase in %crude protein observed where there is high microbial load (i.e., in those fruits stored in the room and in water) (Fig. 1). The high difference in the %crude protein shows that microorganisms made significant contribution to the deterioration of breadfruit.

**Table 2:** Microbial load of breadfruit stored under different storage conditions

Storage	At start	In room at 28 ± 2°C (cfu/mL)	In water at20 ± 2°C (cfu/mL)	In refrigerator at 10 ± 2°C	In freezer at −6 ± 2°C	In vinegar, 10% acetic acid (cfu/mL)
Bacterial load	Nil^a^	1.6 × 10^1b^	1.1 × 10^1b^	Nil^a^	Nil^a^	0.02 × 10^1a^
Fungal load	Nil^a^	7.2 × 10^1c^	5.3 × 10^1d^	Nil^a^	Nil^a^	0.04 × 10^1a^

Means with different superscripts within the same row are significantly different (*P* ≤ 0.05), *n *=* *4.

**Table 3:** Morphological changes of breadfruit kept under different storage conditions

Storage	0 h					After 120 h				
	Color		Texture		Odor	Color		Texture		Odor
	Pulp	Bark	Pulp	Bark		Pulp	Bark	Pulp	Bark	
In room at 28 ± 2°C	White	Green	Hard	Rough	None	Black	Black	Spongy	Rough	Putrefying
In water at 20 ± 2°C	White	Green	Hard	Rough	None	Yellowish green	Black	Very soft	Spongy	Putrefying
In refrigerator at 10 ± 2°C	White	Green	Hard	Rough	None	Yellow	Black	Very soft shriveled	Shriveled	None
In freezer at −6 ± 2°C	White	Green	Hard	Rough	None	Yellow	Black	Collapsed	Shriveled	None
In vinegar (10% acetic acid)	White	Green	Hard	Rough	None	Yellow	Yellow	Spongy	Spongy	None

## Conclusion

Although the African breadfruit (*A. communis*, Forst) deteriorates due to its high respiration rate in storage, this study has revealed that deterioration rate becomes significantly higher with presence of spoilage microorganisms. Provision of good storage conditions will substantially delay spoilage of breadfruit, the knowledge of which will contribute essentially to nutrition and economy of the Nigerian populace.

## Conflict of Interest

None declared.
